# Suprapatellar intramedullary nailing of tibial shaft fractures in pregnancy. A report of two cases

**DOI:** 10.1186/s12884-022-04835-4

**Published:** 2022-06-28

**Authors:** Attilio Basile, Laura Palmieri, Riccardo Lanzetti, Pasquale Sessa, Marco Spoliti, Alessio Giai Via, Gennaro Pipino

**Affiliations:** 1 Department of Orthopedic Surgery and Traumatology, San Camillo-Forlanini Hospital, Rome, Italy; 2 Department of Orthopedic Surgery and Traumatology, Policlinico Tor Vergata, School of Medicine, University of Tor Vergata, Rome, Italy; 3 School of Medicine, University of Salerno, Salerno, Italy; 4 UCM Malta University, campus Lugano, Lugano, Switzerland; 5 Department of Orthopedic Surgery and Traumatology, Villa Erbosa Hospital, Gruppo San Donato, Bologna, Italy

**Keywords:** Tibial fracture, Intramedullary nailing, Suprapatellar surgical approach, Pregnancy, Pregnancy trauma

## Abstract

**Background:**

Treatment of closed tibial shaft fractures in the 3^rd^ trimester of pregnancy is controversial. Since there are few case reports published in literature, there is no consensus on the appropriate management of these fractures. This case report proposes intramedullary nailing throught the suprapatellar approach for the treatment of tibial shaft fracture in pregnant women, never described before in literature.

**Case presentation:**

We report 2 cases of a tibial diaphyseal fracture treated by intramedullary nailing in women at the 3^rd^ trimester of pregnancy.

**Conclusion:**

Surgical treatment of tibial shaft fracture of pregnant women in the 3^rd^ trimester of pregnancy with intramedullary nailing seems to be safe. The use of the specific suprapatellar approach helps in the intra-operative management of the pregnant patients.

## Background

Tibial diaphyseal fractures are common fractures of the long bones, and account for approximately 2% of all fractures [1]. Although closed reduction and intramedullary nail fixation is the gold standard treatment for closed tibial shaft fracture [2], it is unclear which is the standard treatment in pregnant women.

Intramedullary nailing is a minimally invasive surgery which preserves soft tissues, retains the periosteal blood support, and provides high union rates with a low rate of complications, enhances fracture healing and clinical outcomes [3]. Furthermore, early weight-bearing and fast limb mobilization reduce both the risk of deep venous thrombosis (DVT) and secondary pulmonary embolism, which are higher in pregnancy due to a pregnancy-induced prothrombotic state [4]. Therefore, the surgical treatment of tibia diaphyseal fractures and the use of intramedullary nailing seem to be the best treatment also for pregnant women; however, poor evidence is available on this topic, mainly limited to few case reports. Furthermore, the anesthesia procedures and radiation exposure associated with surgery are a common matter of concerns for both the mother and the orthopedic surgeon. The management of long bone fractures in pregnancy, therefore, should be evaluated by a multidisciplinary team, to assess all the possible risks, and to support the staff and the patient in making an informed choice.

In this article we present the cases of 2 pregnant women at the 36^th^ and 32^nd^ weeks of pregnancy, who suffered a tibial shaft fracture, that were surgically treated by an intramedullary nailing through a suprapatellar approach. To the best of our knowledge, no other cases of tibial fractures treated with an intramedullary nail using a suprapatellar approach have ever been described in pregnancy.

## Case presentation

### Case 1

**Fig. 1:**
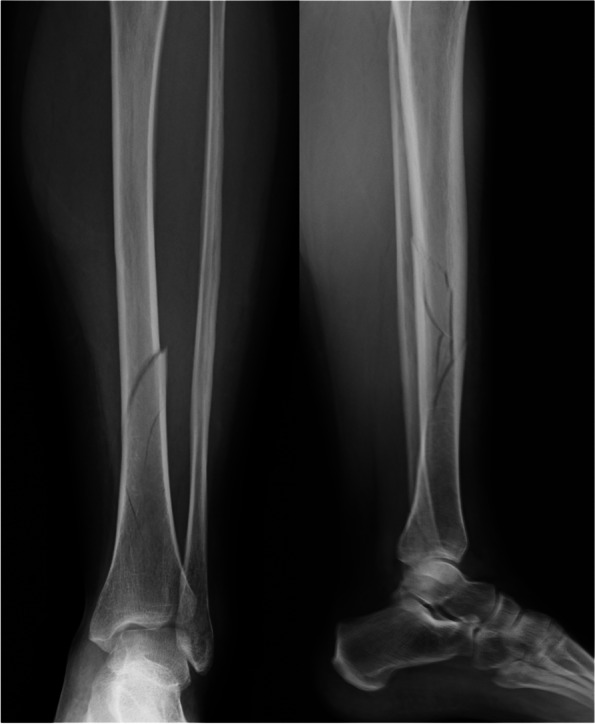
Preoperatory X-ray of the left leg

A 36-year-old 36 weeks pregnant woman came to the Emergency Department of San Camillo-Forlanini Hospital in Rome due to an accidental fall while she was walking near her home, with consequent trauma of the left leg. The patient was examined by both an orthopedic surgeon and a gynaecologist and received the appropriate radiological assessments. The orthopedic physical examination did not reveal any peripheral nerve or vascular deficits, nor compartment syndrome; however, the left leg appeared swollen and painful at palpation. The standard two view X-ray examination of the left tibia documented a 42-A1 tibial diaphyseal fracture, and 44-B1 distal fibula fracture according to the AO-classification (Fig. 1). The fractures were initially immobilized with a long femoro-podalic cast, which was poorly tolerated by the patient.

**Fig. 2:**
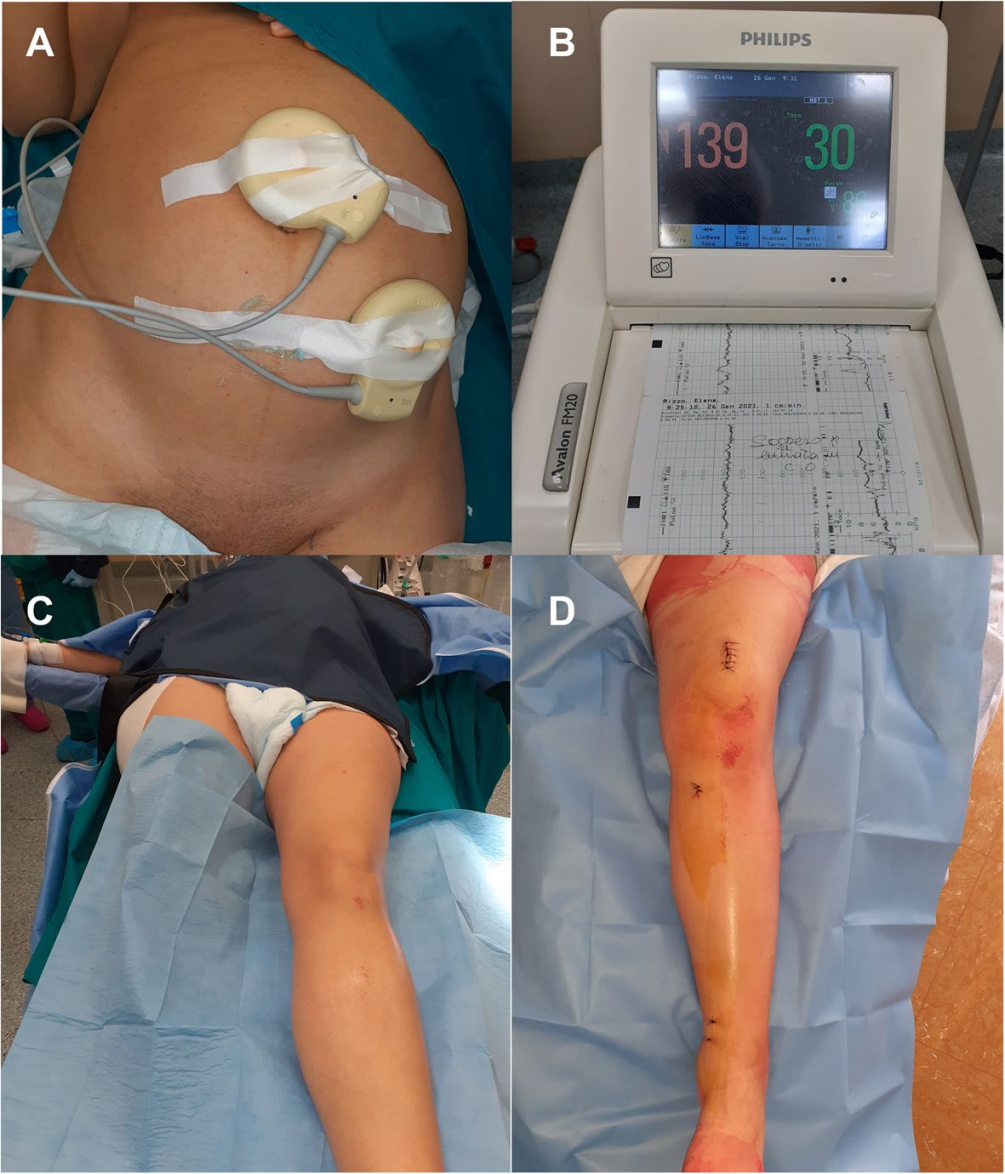
Intraoperatory images and surgical approaches. **A** The fetus was monitored throughout all the surgery with continuous CTG, in order to detect any possible anomalies to the fetus. **B** intraoperatory record of the CTG. **C** A lead shield was placed over the patient's abdomen to protect the fetus against to intraoperatory radiations. **D** Postoperatory image showing the suprapatellar surgical approach

The gynecological examination was unremarkable for both the fetus and the mother, and no restrictions on surgery were raised. A written informed consent was obtained by the patient. A gynecologist was present during the entire surgical procedure in case an emergent caesarean delivery was necessary. The fetus was monitored throughout the surgical procedure with a continuous cardiotocography (CTG). A loco-regional anesthesia was performed, and the patient was placed supine on a standard operating table. A wedge was placed under the right buttock to tilt the spine, reduce the pressure on the Aorta and inferior vena cava, and to reduce the risk of aortocaval compression syndrome. A lead shield was placed over the patient's abdomen to minimize the radiation dose absorbed by the fetus (Fig. 2). The lower limb was prepped and draped in the usual sterile fashion, and surgery was performed without the need of a tourniquet.

**Fig. 3:**
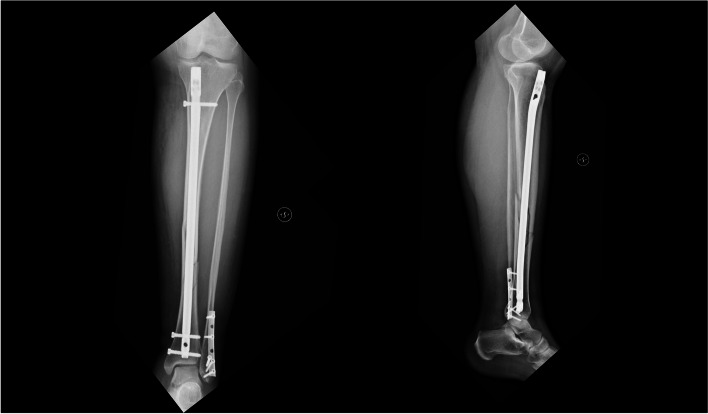
Postoperatory X-ray

According to surgical technique, a 3-cm longitudinal skin incision was made 2 cm above the proximal pole of the patella. The quadriceps tendon was exposed by blunt dissection, a longitudinal midline incision was made. The guide wire was introduced into the knee joint, the correct entry point was checked and confirmed under fluoroscopy in both planes. The fracture was provisionally reduced, and the medullary canal reamed. A T2 tibia nail (Stryker®, Kalamazoo, USA) was introduced to fix the tibial fracture. During surgery, the distal fibula fracture was also evaluated, which was not displaced, and the length of the fibula was preserved. The osteosynthesis with plate and screws was performed (Fig. 3).

Low molecular weight heparin (LMWH) was administered as prophylaxis for thromboembolism (4.000 IU Enoxaparin/day). A boot was prescribed for 30 days due to concomitant fracture of the distal fibula, and the patient was mobilized with the aid of two crutches since the first postoperative day. She was discharged two days after surgery. Isometric muscular strength exercises and passive and active mobilization of the knee and the ankle were indicated to gain a full recovery of the knee range of motion. There were no complications during follow-up period. Two weeks after surgery, at the 38 week the patient gave birth to a healthy newborn. The delivery was elective cesarean section for reasons not related to the fracture or surgery.

**Fig. 4:**
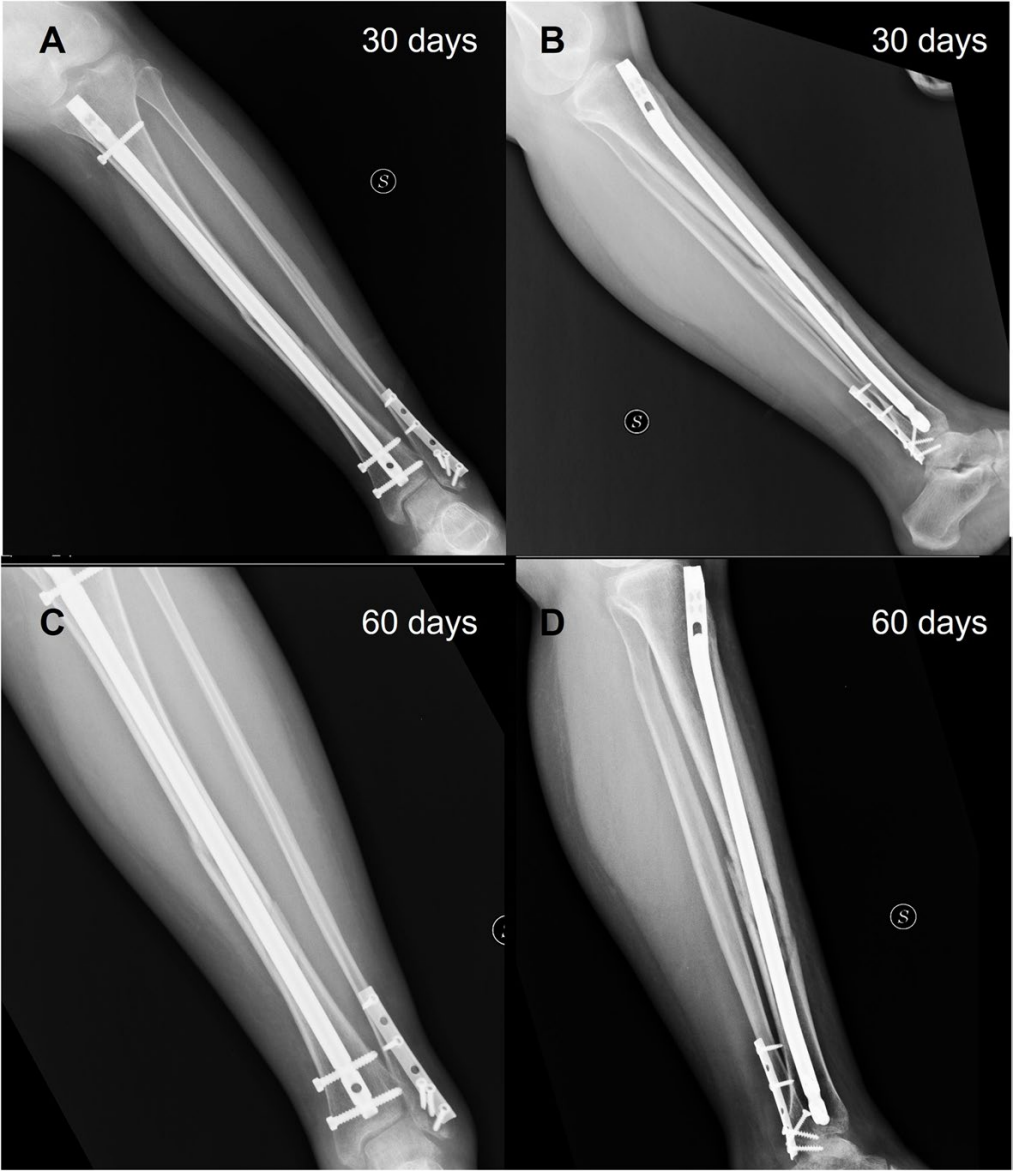
Control plain radiographs at 30 days postop. (**A** and **B**) and at 60 days follow-up (**C** and **D**)

**Fig. 5:**
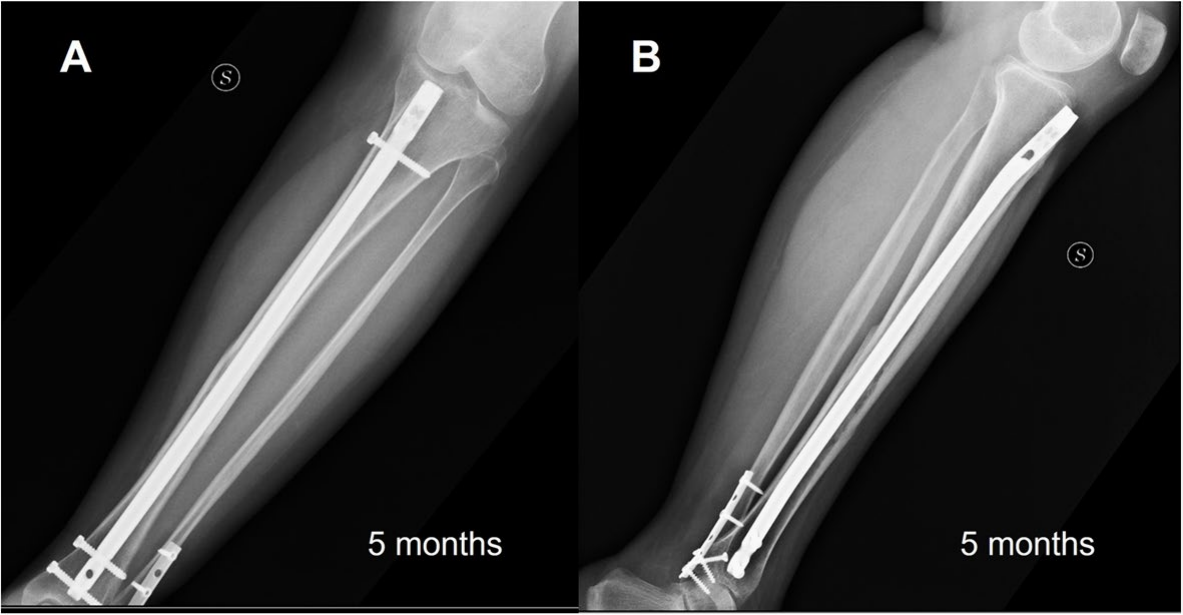
Plain radiograph of the left leg at 5 months follow-up

**Fig. 6:**
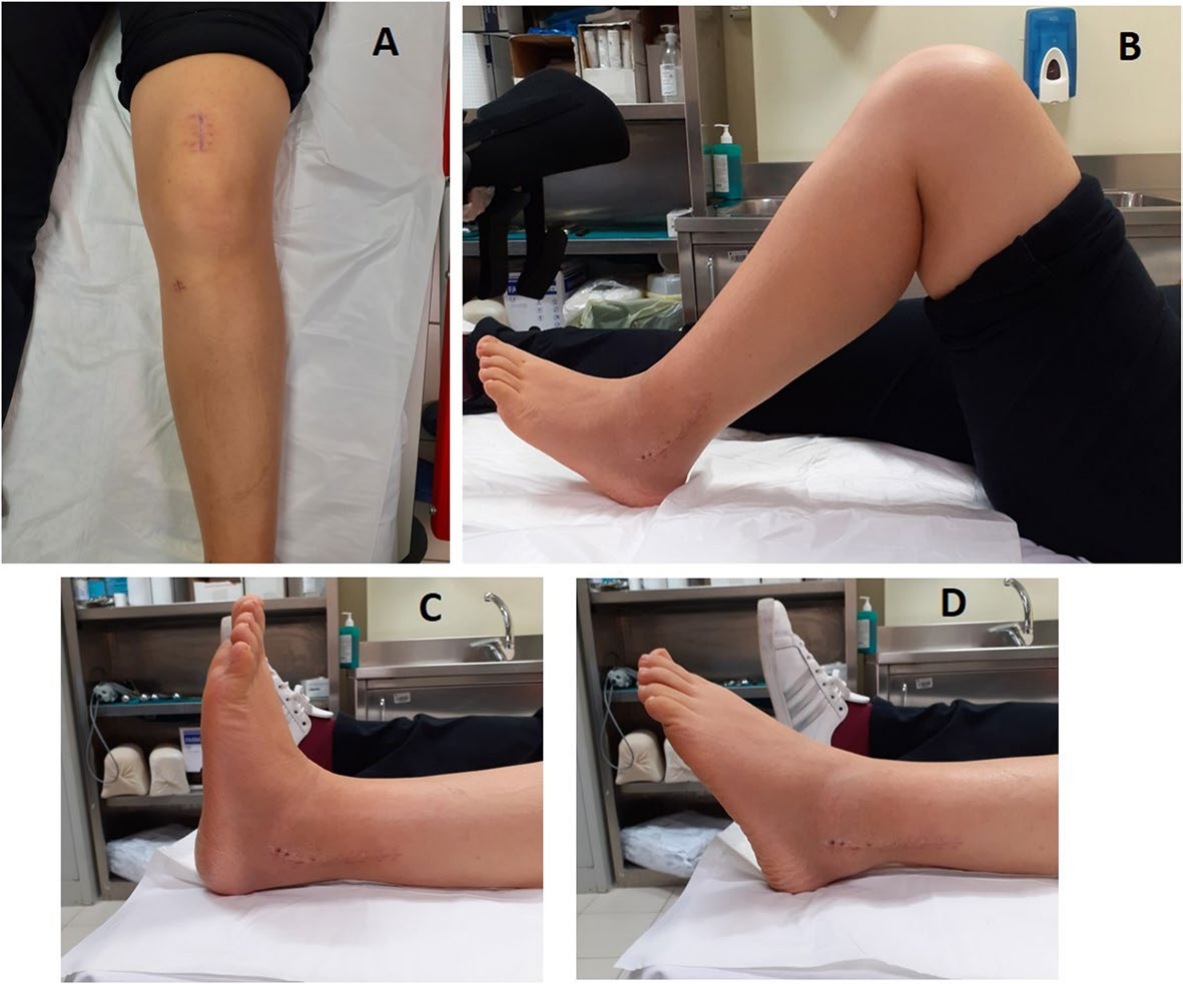
Clinical images at final follow-up. The scars are in order, without signs of local inflammation. The Range of Motion of the knee and ankle are complete (Figures **A**, **B**, **C** and **D**)

Thirty days after surgery the patient was able to walk with 2 crutches, weight bearing as tolerated, and started a physiotherapy program to recover muscular strength, and proprioception. Two months after surgery weight bearing with one crutch was allowed, and the patients continued muscle strengthening and exercises to restore proprioception. Three months after the surgery, she was able to walk without crutches. Post-operative radiographs at 30, 60 days and 5 months (Figs. 4 and 5) documented excellent healing of both the fractures of the tibia and fibula. The patient did not experience complication in the follow up (Fig. 6). She reported some pain at her left knee, that resolved spontaneously 4 months after surgery.

### Case 2

This is the case of a 33-year-old woman at 32^nd^ weeks of pregnancy, who was hospitalized for a 42-A1 tibial shaft fracture of her right leg. The patient was treated with suprapatellar intramedullary nailing. A written informed consent was signed by the patient. No adverse events have been reported during the follow-up period, and at the 40^th^ week of pregnancy she gave birth a healthy boy. The delivery was vaginal spontaneous.

The pre- and post-operatory program were the similar as applied for the previous case. The patient was allowed to start passive and active mobilization of the knee and ankle, as she did not sustain an associated distal fibula fracture, and started isometric strengthening exercises since the second day post surgery. Walking with 2 crutches and 10 kg weight bearing were allowed the day after surgery, and at thirty days after surgery the patient was able to walk with weight bearing as tolerated. Muscle strengthening program and proprioception exercises were the same as in the previous patient. The 5 months X-ray follow-up showed the healing of the tibial shaft fracture.

## Discussion and conclusion

The management of fractures in pregnancy is challenging. The indications for the appropriate management are unclear, and surgical treatment is a difficult choice, both for the mother and the orthopedic surgeon, mainly due to the theoretical associated risks. For this reason, a multidisciplinary team assessment of the case is recommended to evaluate all the possible risks due to anesthetic agents, anticoagulants, patient’s intraoperatory position, and radiation exposure. A risk/benefit evaluation is of primary importance to ensure the most appropriate treatment.

After a careful meeting, we believed that benefits deriving from the surgical treatment overcome the ones of the conservative treatment for our patients, also considering that during pregnancy the raise of estrogen determine a more rapid fracture healing [5]. Conservative management of a tibial shaft fracture in adult patient consists in immobilization in a high femoro-podalic cast (from the thigh to the foot, with the knee at 20° of flexion) for 30–40 days, then a below knee cast for other 30–60 days. Loss of reduction and malagninemts are common complications of conservative treatment, which are not acceptable in young patients. Furthermore, the movements of the lower limb are extremely reduced because of the cast, making more difficult the positioning of the patient during delivery, both vaginal and cesarean. Surgical stabilization of the tibial shaft fracture reduces pain, and improves function and movements of the injured limb after surgery. The patient can be discharge the day after or 2 days after surgery, with minimum pain and without a cast or brace. The operated limb can be moved without restrictions, and placed in the correct position during the delivery. Therefore, surgical treatment not only do not affect the mode of delivery, but it can facilitate a vaginal spontaneous delivery.

We analyzed all the possible risks and, to keep those at minimum, the entire team took specific actions. Although general anesthesia is generally safe during pregnancy, the anesthesiologist selected the locoregional anesthesia: the fetus was thus less exposed to medications [6], and the risk of fetal hypoxia and preterm delivery is reduced [7]. Also, the position of the patient on the operating table was carefully considered to reduce the risk of aortocaval compression syndrome. This syndrome may occur during the second and third trimester of pregnancy due to the compression of the abdominal aorta and inferior vena cava by the enlarged uterus and, consequently, it may cause the reduction of both the cardiac output and the placentar perfusion [8]. To minimize the risk of developing such syndrome, ideally the left-lateral decubitus position should be considered. However, since this would have not been convenient for the surgical procedure, as an alternative we decided to place a 10 cm wedge under the right gluteus in order to tilt the spine by 15 degrees, and move the uterus sideways [9, 10].

Moreover, another concern generally associated with pregnant women arises from their typical state of hypercoagulability [11], since this could lead to the development of DVT and thromboembolism during immobilization after trauma. Intramedullary nailing allows early mobilization of the patients, reducing the risk of DVT together with LMWH. LMWH has also got the advantage to not cross the placenta and thus not to damage the fetus [10, 11].

The team evaluated the risk of malformations in the fetus resulting from radiological exposure. Generally, this risk appears to be high during the period between the third and fifteenth week of gestation, when the central nervous system is developing, and then significantly reduces as pregnancy progresses [12]. Considering that the American National Council on Radiation Protection and Measurements prescribes that the maximum cumulative dose of radiation to which a foetus may be exposed shall not exceed 5 rads [13, 14], we took all necessary measures to minimize the radiological risk. However, in our case report the patients were at the 36^th^ and 32^nd^ week of pregnancy, therefore we were reasonably certain that the use of X-ray in pre-operative planning and fluoroscopy during surgery would have not endangered the fetus growing. Nonetheless, the use of a cardiotocography and the ultrasounds allowed to closely monitor the fetus conditions throughout the surgical procedure.

For the surgery, we opted for an intramedullary nailing as this is the gold standard treatment according to the current literature [2]. Specifically, unlike the works of Walsh [15] and Bozkurt [16], which presented the common infra-patellar incision, we selected for the supra-patellar surgical approach. The literature is rich in works which describe the multiple advantages of this approach for the tibial shaft fractures [17, 18]. The suprapatellar intramedullary nailing seems to reduce blood loss, and thus the consequent demand for post-operative transfusions [19]. The two most common causes of anemia in pregnancy and the puerperium are iron deficiency and acute blood loss. Acute anemia has been associated with abnormal fetal oxygenation, therefore it is important to reduce perioperatory bleeding [20]. The fluoroscopy time was also minimized, reducing the exposure to ionizing radiation of the fetus [21, 22]. The knee can be placed in the semi-extended position on the operating table, and the radiologist can easily make the right projections during surgery; in this way the surgeon will quickly find the correct entry point minimizing the amount of shots required [23].

Suprapatellar approach has been reported to reduce post-operative and anterior knee pain compared to standard trans-patellar tendon approach [24]. The absence of incision and dissection near the infrapatellar area seems to protect the infrapatellar branches of the saphenous nerve, resulting in a significant reduction of post-operative pain [18, 25]. Postoperatory pain is mild, and it usually resolves in 7–10 days after surgery. The second patient was pain free at the moment of the childbirth, while the first patient experienced a moderate knee pain (VAS score 4), which resolved after 4 months, and it did not affect the mode of delivery.

Lastly, in the intramedullary nailing the malalignment of proximal tibial fragments is among the most fearsome complications. In particular, when performing the intra-patellar approach, this can happen following the flection of the knee: the extensor complex tends to cause a displacement of the proximal fragment. Conversely, the supra-patellar approach and the supine intraoperatory position reduce the risk for malalignment, as the knee remains in a semiextended position throughout the surgery, and this promotes a stable alignment [18, 22, 23, 26].

In literature, only few articles describe the treatment of long bone fracture in a pregnant woman. Specifically, only four articles reported on the management of a tibial shaft fracture (case reports). Two patients were treated surgically [19, 20], in one case surgery was postponed surgery after childbirth [7], and the last patient was treated conservatively with good outcome [27]. No adverse events have been reported by the authors. In this article, we reported good results in terms of fracture healing and clinical outcome after intramedullary nailing of a tibial shaft fracture in a pregnant patient, preserving, at the same time, the health of both the mother and the fetus by reducing radiation exposure and blood loss. The suprapatellar approach appears to be easy and safe in pregnant patients. However, we cannot draw a definitive conclusion on this topic, as this article describes only two cases.

A limitation of the study is the small sample, a report of two cases, which do not allow us to expand our conclusions to the general pregnant population, but this injury is rare in pregnancy. Furthermore, we do not have access to the obstetrics medical records, therefore we don’t have any further detailed information about arrangement and management for delivery, except that reported by the two patients.

In conclusion, suprapatellar intramedullary nailing of tibial shaft fractures seems to be a safe and effective treatment for women during pregnancy. The success of this surgery appears to be mainly linked to two key factors. The first was the employment of a multidisciplinary team which carefully analyzed risks and benefits, identifying a proper solution capable of treating the bone damage and, safeguarding the health of the mother and the fetus. The second key factor was related to the choice of the supra patellar approach, which proved to be an extremely valid solution in managing the alignment of the fracture and the containment of post-operative pain, minimizing, fluoroscopy time and the need for transfusions. However, as this is the report of only 2 cases, more studies are required to confirm these results.

## Data Availability

Please contact the contact the corresponding Author (AGV) for any required supporting data.

## Abbreviations

DVT: Deep Venous Thrombosis
CTG: Cardiotocography
LMWH: Low Molecular Weight Heparin
